# Cationic gold(I) axially chiral biaryl bisphosphine complex-catalyzed atropselective synthesis of heterobiaryls

**DOI:** 10.3762/bjoc.7.105

**Published:** 2011-07-06

**Authors:** Tetsuro Shibuya, Kyosuke Nakamura, Ken Tanaka

**Affiliations:** 1 Department of Applied Chemistry, Graduate School of Engineering, Tokyo University of Agriculture and Technology, Koganei, Tokyo 184-8588, Japan

**Keywords:** asymmetric catalysis, axial chirality, gold, heterobiaryls, hydroarylation

## Abstract

It has been established that a cationic gold(I)/(*R*)-DTBM-Segphos or (*R*)-BINAP complex catalyzes the atropselective intramolecular hydroarylation of alkynes leading to enantioenriched axially chiral 4-aryl-2-quinolinones and 4-arylcoumarins with up to 61% ee.

## Introduction

**Scheme 1:**
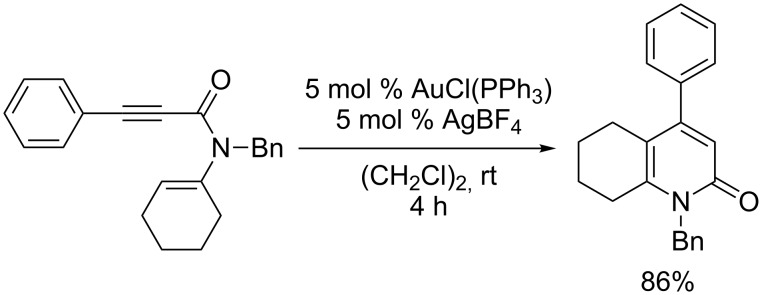
Cationic gold(I)/PPh_3_-complex catalyzed intramolecular hydroalkenylation of alkynes.

Atropselective biaryl synthesis [1–4] has attracted significant interest due to its great utility in asymmetric catalysis and natural product synthesis. In 2004, three research groups, including ours, independently reported transition-metal catalyzed asymmetric [2 + 2 + 2] cycloaddition reactions to produce axially chiral biaryls [5–7]. These reports clearly demonstrated the utility of the asymmetric annulation strategy for the atropselective biaryl synthesis [8]. As an alternative asymmetric annulation method for the atropselective biaryl synthesis, we turned our attention to transition-metal catalyzed hydroalkenylation and hydroarylation reactions [9–15]. In this context, our research group developed the cationic gold(I)/PPh_3_-complex catalyzed intramolecular hydroalkenylation of *N*-alkenyl-arylethynylamides leading to 4-aryl-2-pyridones (Scheme 1) [16–17].

**Scheme 2:**
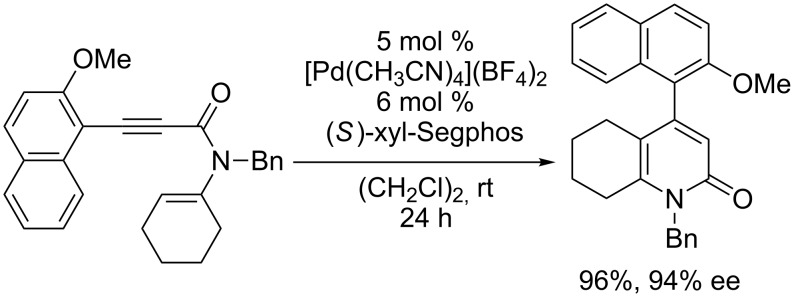
Cationic palladium(II)/(*S*)-xyl-Segphos-complex catalyzed atropselective intramolecular hydroalkenylation of alkynes.

The atropselective synthesis of 6-aryl-2-pyridones has already been achieved by rhodium catalyzed [2 + 2 + 2] cycloaddition [18], while the atropselective synthesis of 4-aryl-2-pyridones has not yet been realized. The application of this intramolecular hydroalkenylation reaction to the atropselective synthesis of 4-aryl-2-pyridones from *N*-alkenyl-arylethynylamides was thus investigated. Although cationic gold(I)/axially chiral biaryl bisphosphine complexes [19–31] have been frequently employed in asymmetric variants of cationic gold(I) catalyses [32–38], including 6-*endo*-*dig* and 6-*exo*-*dig* cyclizations [39–41], the use of these gold(I) complexes gave almost racemic products [42]. Fortunately, cationic palladium(II)/axially chiral biaryl bisphosphine complexes were found to be effective catalysts, and a cationic palladium(II)/(*S*)-xyl-Segphos complex showed the highest enantioselectivity (Scheme 2) [42].

**Scheme 3:**
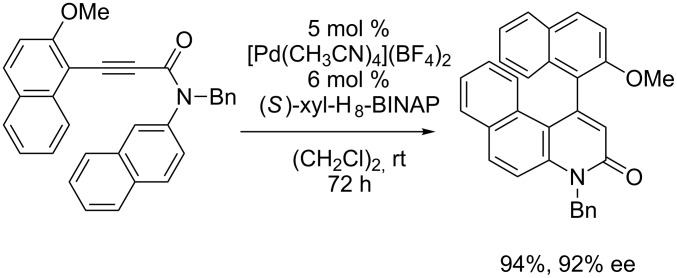
Cationic palladium(II)/(*S*)-xyl-H_8_-BINAP complex-catalyzed atropselective intramolecular hydroarylation of alkynes.

In addition, the cationic palladium(II)/axially chiral biaryl bisphosphine complexes were able to catalyze the asymmetric intramolecular hydroarylation of *N*-aryl-arylethynylamides leading to axially chiral 4-aryl-2-quinolinones, and the cationic palladium(II)/(*S*)-xyl-H_8_-BINAP complex showed the highest enantioselectivity (Scheme 3) [43–44].

In this paper, we report the use of the cationic gold(I)/axially chiral biaryl bisphosphine complexes in the catalytic asymmetric intramolecular hydroarylation for the synthesis of axially chiral 4-aryl-2-quinolinones and 4-arylcoumarins.

## Results and Discussion

**Figure 1:**
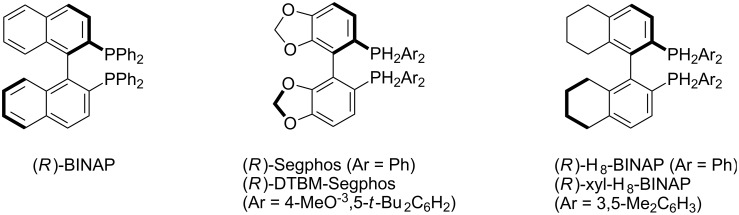
Structures of axially chiral biaryl bisphosphine ligands.

The reaction of *N-*benzyl-*N*-(2-naphthyl)propiolamide **1a**, bearing a 2-methoxynaphthyl group at an alkyne terminus, was first investigated in the presence of a cationic gold(I)/(*R*)-BINAP complex (20 mol % Au). Although the reaction proceeded at room temperature in good yield, enantioselectivity was low (Table 1, entry 1). The effect of axially chiral biaryl bisphosphine ligands (Figure 1) on the yield and the enantioselectivity was then investigated. Among the bis(diphenylphosphine) ligands examined (Table 1, entries 1–3), the use of (*R*)-H_8_-BINAP furnished **2a** with the highest enantiomeric excess (Table 1, entry 3). An increase in the steric bulk of the aryl group on the phosphorus atom of H_8_-BINAP lead to a decrease in the ee (Table 1, entry 4). The use of sterically more demanding (*R*)-DTBM-Segphos as a ligand furnished **2a** in high yield with the highest ee (Table 1, entry 5). Unfortunately, a reduction in the amount of gold to 10 mol % significantly decreased both product yield and enantioselectivity (Table 1, entry 6).

**Table 1:** Screening of axially chiral biaryl bisphosphine ligands for the cationic gold(I)-complex catalyzed atropselective intramolecular hydroarylation of **1a**.^a^

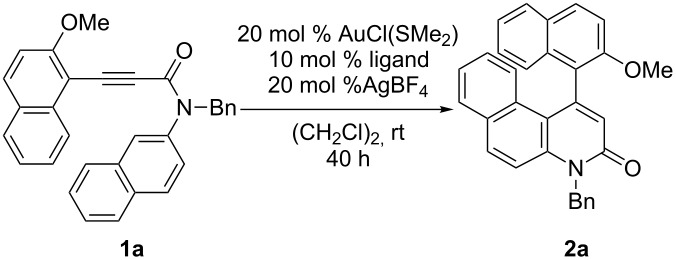				

Entry	Ligand	Convn (%)^b^	Yield (%)^c^	ee (%)

1	(*R*)-BINAP	81	75	7 (*S*)
2	(*R*)-Segphos	90	81	8 (*R*)
3	(*R*)-H_8_-BINAP	94	93	17 (*S*)
4	(*S*)-xyl-H_8_-BINAP	100	96	13 (*R*)
5	(*R*)-DTBM-Segphos	100	96	59 (*R*)
6^d^	(*R*)-DTBM-Segphos	71	49	31 (*R*)

^a^AuCl(SMe_2_) (0.010 mmol, 20 mol %), AgBF_4_ (0.010 mmol, 20 mol %), ligand (0.0050 mmol, 10 mol %), **1a** (0.050 mmol), and (CH_2_Cl)_2_ (1.5 mL) were used. ^b^Determined by ^1^H NMR. ^c^Isolated yield. ^d^AuCl(SMe_2_) (0.010 mmol, 10 mol %), AgBF_4_ (0.010 mmol, 10 mol %), ligand (0.0050 mmol, 5 mol %), **1a** (0.10 mmol), and (CH_2_Cl)_2_ (1.5 mL) were used. Reaction time: 72 h.

Thus, the scope of the cationic gold(I)-complex catalyzed atropselective intramolecular hydroarylation of alkynes was explored at room temperature, as shown in Table 2. The 2-methoxynaphthalene derivative **1a** (Table 2, entry 1) and the 2-methoxymethoxynaphthalene derivative **1b** (Table 2, entry 2) furnished the desired benzoquinolinones **2a** and **2b**, respectively, in high yields and high ee, using (*R*)-DTBM-Segphos as a ligand. In addition, benzocoumarin **2c** (Table 2, entry 3) was obtained in moderate ee, although the yield was low due to partial deprotection of the methoxymethoxynaphthalene moiety (Table 2, entry 3). The reactions of carbazole and dialkoxybenzene derivatives **1d**–**g**, using (*R*)-DTBM-Segphos as a ligand, furnished the corresponding quinolinone and coumarin derivatives **2d**–**g** in high yields with perfect regioselectivity, while the observed ee values were very low (<10% ee). However, interestingly, the use of (*R*)-BINAP as a ligand improved the enantoselectivity (14–32% ee, Table 2, entries 4–7).

**Table 2:** Cationic gold(I)-complex catalyzed atropselective intramolecular hydroarylation of **1a**–**g** leading to heterobiaryls **2a**–**g**.^a^

Entry	**1**		Ligand (time)	**2**		% yield^b^ (% ee)

1	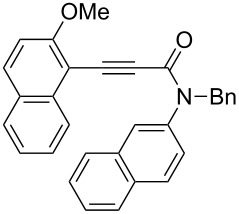	**1a**	(*R*)-DTBM-Segphos (40 h)	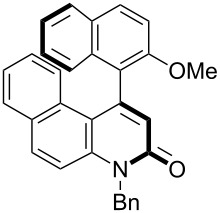	(*R*)-(−)-**2a**	96 (59)
2	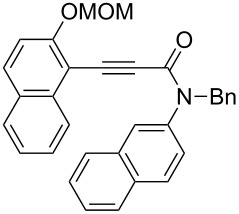	**1b**	(*R*)-DTBM-Segphos (72 h)	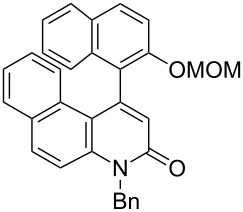	(−)-**2b**	87 (61)
3	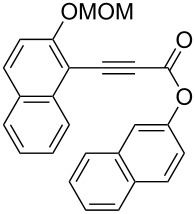	**1c**	(*R*)-DTBM-Segphos (40 h)	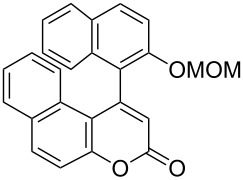	(−)-**2c**	33 (49)
4	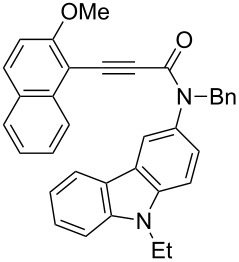	**1d**	(*R*)-BINAP (72 h)	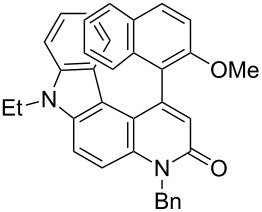	(+)-**2d**	82 (28)
5	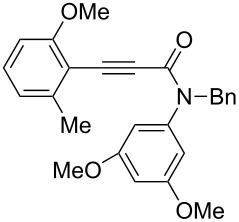	**1e**	(*R*)-BINAP (40 h)	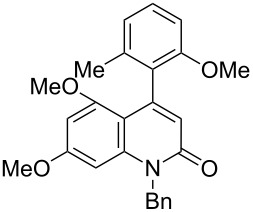	(−)-**2e**	100 (32)
6	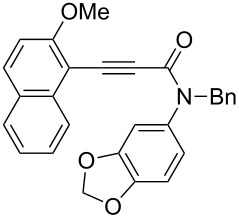	**1f**	(*R*)-BINAP (40 h)	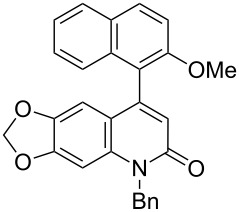	(+)-**2f**	88 (27)
7	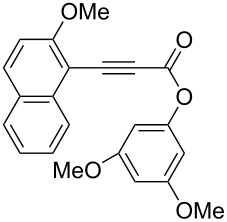	**1g**	(*R*)-BINAP (40 h)	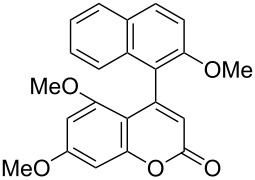	(+)-**2g**	93 (14)

^a^Reactions were conducted using AuCl(SMe_2_) (0.010 mmol), AgBF_4_ (0.010 mmol), (*R*)-DTBM-Segphos or (*R*)-BINAP (0.0050 mmol), **1a**–**g** (0.050 mmol), and (CH_2_Cl)_2_ (1.5 mL) at rt. In all entries, 100% convn of substrates **1a**–**g** was observed. ^b^Isolated yield.

**Scheme 4:**
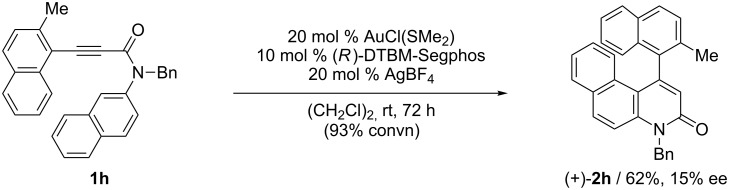
Cationic gold(I)/(*R*)-DTBM-Segphos-complex catalyzed atropselective intramolecular hydroarylation of 2-methylnaphthalene derivative **1h**.

In the previously reported cationic palladium(II)/(*S*)-xyl-H_8_-BINAP-complex catalyzed atropselective intramolecular hydroarylation of alkynes, the presence of the 2-methoxy-substituted aryl group at the alkyne terminus was important for the realization of both high reactivity and enantioselectivity [40]. Similarly, the reaction of 2-methylnaphthalene derivative **1h** in the presence of the cationic gold(I)/(*R*)-DTBM-Segphos complex furnished the corresponding benzoquinolinone **2h** with lower yield and ee than those of **2a** (Scheme 4).

## Conclusion

In conclusion, it has been established that a cationic gold(I)/(*R*)-DTBM-Segphos or (*R*)-BINAP complex catalyzes the atropselective intramolecular hydroarylation of alkynes leading to enantioenriched axially chiral 4-aryl-2-quinolinones and 4-arylcoumarins in up to 61% ee. Although there clearly remains room for improvement in enantioselectivity, the present asymmetric catalysis is a rare example of the utilization of gold(I)/chiral phosphine catalysts for the construction of noncentrochirality [45–47].

## Experimental

**General: **^1^H NMR spectra were recorded at 300 MHz (JEOL AL 300). ^13^C NMR spectra were obtained with complete proton decoupling at 75 MHz (JEOL AL 300). HRMS data were obtained on a Bruker micrOTOF Focus II. Infrared spectra were obtained on a JASCO FT/IR-4100. Optical rotations were obtained on a JASCO DIP-1000. Melting points were obtained on a METTLER MP50. Anhydrous (CH_2_Cl)_2_ (No. 28,450-5) was purchased from Aldrich and used as received. Solvents for the synthesis of substrates were dried over molecular sieves (4 Å, Wako) prior to use. Substrates **1a**, **1b**, **1d**, **1e**, **1f**, and **1h** were prepared according to the literature [43]. Products **2a**, **2b**, **2d**, **2e**, **2f**, and **2h** were already reported [43]. All other reagents were obtained from commercial sources and used as received. All reactions were carried out under an atmosphere of argon or nitrogen in oven-dried glassware with magnetic stirring.

**(2-Methoxymethoxynaphthalen-1-yl)propynoic acid naphthalen-2-yl ester (1c):** To a stirred solution of 3-[2-(methoxymethoxy)-1-naphthalenyl]-2-propynoic acid [48] (0.256 g, 1.00 mmol), 2-naphthol (0.159 g, 1.10 mmol), and 4-dimethylaminopyridine (12.2 mg, 0.100 mmol) in CH_2_Cl_2_ (10 mL) was added a solution of dicyclohexylcarbodiimide (0.248 g, 1.20 mmol) in CH_2_Cl_2_ (3 mL) at 0 °C, and the mixture was stirred at 0 °C for 2 h and at room temperature for 18 h. The crude mixture was filtered with CH_2_Cl_2_. The filtrate was washed with brine, dried over Na_2_SO_4_, and concentrated. The residue was purified by a silica gel column chromatography (hexane/EtOAc = 10:1) to give **1c** (0.222 g, 0.580 mmol, 58% yield). Yellow solid; mp 97.3–99.3 °C; IR (KBr): 2203, 1717, 1229, 1149, 1005 cm^−1^; ^1^H NMR (CDCl_3_, 300 MHz) δ 8.15–8.01 (m, 1H), 7.97–7.70 (m, 6H), 7.61–7.31 (m, 6H), 5.36 (s, 2H), 3.56 (s, 3H); ^13^C NMR (CDCl_3_, 125 MHz) δ 160.0, 152.8, 148.1, 134.7, 133.7, 133.4, 131.7, 129.7, 128.8, 128.3, 127.82, 127.79, 126.7, 126.0, 125.1, 124.8, 121.0, 118.8, 115.5, 103.8, 95.1, 89.1, 85.2, 56.6; HRMS–ESI (*m*/*z*): [M + Na]^+^ calcd for C_25_H_18_O_4_Na, 405.1097; found, 405.1107.

**(2-Methoxynaphthalen-1-yl)propynoic acid 3,5-dimethoxyphenyl ester (1g):** The title compound was prepared from (2-methoxynaphthalen-1-yl)propynoic acid [49] and 3,5-dimethoxyphenol in 70% yield by the procedure used for **1c**. Yellow solid; mp 102.9–104.7 °C; IR (KBr): 2211, 1714, 1621, 1269, 1156 cm^−1^; ^1^H NMR (CDCl_3_, 300 MHz) δ 8.16 (d, *J* = 8.4 Hz, 1H), 7.96 (d, *J* = 9.2 Hz, 1H), 7.80 (d, *J* = 8.1 Hz, 1H), 7.59 (dd, *J* = 8.1, 6.5 Hz, 1H), 7.42 (dd, *J* = 8.4, 6.5 Hz, 1H), 7.26 (d, *J* = 9.2 Hz, 1H), 6.43 (d, *J* = 2.1 Hz, 2H), 6.40 (t, *J* = 2.1 Hz, 1H), 4.06 (s, 3H), 3.80 (s, 6H); ^13^C NMR (CDCl_3_, 125 MHz) δ 162.1, 161.1, 152.5, 151.8, 135.0, 133.6, 128.4, 128.3, 128.1, 124.7, 112.1, 101.8, 100.2, 98.7, 89.3, 85.0, 56.5, 55.5; HRMS–ESI (*m*/*z*): [M + Na]^+^ calcd for C_22_H_18_O_5_Na, 385.1046; found, 385.1047.

**General procedure for cationic gold(I)/axially chiral biaryl bisphosphine complex-catalyzed atropselective intramolecular hydroarylation of *N*-aryl-arylethynylamides 1:** To AuCl(SMe_2_) (0.010 mmol) was added a solution of axially chiral biaryl bisphosphine ligand (0.0050 mmol) in (CH_2_Cl)_2_ (0.5 mL), and the mixture was stirred at room temperature for 1 h. To this solution was added AgBF_4_ (0.010 mmol) in (CH_2_Cl)_2_ (0.5 mL) at room temperature, and the mixture was stirred at room temperature for 0.5 h. To this mixture was added a solution of **1** (0.050 mmol) in (CH_2_Cl)_2_ (0.5 mL) at room temperature. After stirring at room temperature for 40–72 h, the mixture was directly purified on a preparative TLC to afford **2**.

**(−)-1-(2-Methoxymethoxynaphthalen-1-yl)benzo[*f*]chromen-3-one [(−)-2c]:** Colorless solid; mp 169.4–170.8 °C; [α]^25^_D_ −86.1 (*c* 0.28, CHCl_3_, 49% ee); IR (KBr): 1738, 1510, 1244, 1050, 1011 cm^−1^; ^1^H NMR (CDCl_3_, 500 MHz) δ 8.05 (d, *J* = 8.7 Hz, 1H), 8.02 (d, *J* = 9.2 Hz, 1H), 7.92 (d, *J* = 8.4 Hz, 1H), 7.81 (d, *J* = 8.0 Hz, 1H), 7.62 (d, *J* = 8.7 Hz, 1H), 7.57 (d, *J* = 9.2 Hz, 1H), 7.53 (d, *J* = 8.4 Hz, 1H), 7.42 (ddd, *J* = 8.4, 7.0, 1.4 Hz, 1H), 7.36 (ddd, *J* = 8.4, 6.9, 1.5 Hz, 1H), 7.32 (ddd, *J* = 8.0, 7.0, 1.0 Hz, 1H), 7.17 (d, *J* = 8.3 Hz, 1H), 6.97 (ddd, *J* = 8.3, 6.9, 1.4 Hz, 1H), 6.45 (s, 1H), 5.04 (dd, *J* = 22.4, 6.9 Hz, 2H), 3.05 (s, 3H); ^13^C NMR (CDCl_3_, 125 MHz) δ 160.6, 154.8, 152.5, 150.6, 133.8, 131.6, 131.0, 130.9, 129.8, 129.5, 129.0, 128.2, 127.7, 127.6, 125.4, 124.8, 124.4, 123.8, 122.8, 118.6, 117.8, 115.7, 114.2, 94.2, 56.0; HRMS–ESI (*m*/*z*): [M + Na]^+^ calcd for C_25_H_18_O_4_Na, 405.1097; found, 405.1085; CHIRALPAK OD-H, hexane/iPrOH = 80:20, 1.0 mL/min, retention times: 14.3 min (major isomer) and 19.0 min (minor isomer).

**(+)-5,7-Dimethoxy-4-(2-methoxynaphthalen-1-yl)chromen-2-one [(+)-2g]:** Colorless solid; mp 148.8–150.4 °C; [α]^25^_D_ +44.9 (*c* 0.24, CHCl_3_, 14% ee); IR (KBr): 1718, 1618, 1598, 1351, 1114 cm^−1^; ^1^H NMR (CDCl_3_, 300 MHz) δ 7.88 (d, *J* = 9.0 Hz, 1H), 7.85–7.77 (m, 1H), 7.48–7.39 (m, 1H), 7.38–7.27 (m, 3H), 6.57 (d, *J* = 2.4 Hz, 1H), 6.12 (d, *J* = 2.4 Hz, 1H), 6.07 (s, 1H), 3.85 (s, 3H), 3.83 (s, 3H) 3.07 (s, 3H); ^13^C NMR (CDCl_3_, 125 MHz) δ 163.1, 161.2, 158.5, 157.1, 152.4, 151.0, 131.9, 129.3, 128.5, 127.9, 126.6, 124.2, 123.6, 122.9, 114.0, 113.1, 105.0, 95.8, 93.6, 56.7, 55.75, 55.71; HRMS–ESI (*m*/*z*): [M + Na]^+^ calcd for C_22_H_18_O_5_Na, 385.1046; found, 385.1036; CHIRALPAK AD-H, hexane/iPrOH = 80:20, 1.0 mL/min, retention times: 8.8 min (minor isomer) and 10.5 min (major isomer).

## Supporting Information

File 1 ^1^H and ^13^C NMR spectra for new compounds **1c**, **1g**, **2c**, and **2g**.
