# Supplementary material for: Cationic gold(I) axially chiral biaryl bisphosphine complex-catalyzed atropselective synthesis of heterobiaryls
Source: Beilstein J Org Chem. 2011 Jul 6;7:944–50. doi: 10.3762/bjoc.7.105 (PMC3170204; doi:10.3762/bjoc.7.105)

Supporting Information

for

Cationic gold(I) axially chiral biaryl bisphosphine complex-catalyzed atropselective synthesis of heterobiaryls

Tetsuro Shibuya, Kyosuke Nakamura and Ken Tanaka*

Address: Department of Applied Chemistry, Graduate School of Engineering, Tokyo University of Agriculture and Technology, Koganei, Tokyo 184-8588, Japan

Email: Ken Tanaka* - tanaka-k@cc.tuat.ac.jp

*Corresponding author

^{1}H and ^{13}C NMR spectra for new compounds 1c, 1g, 2c, and 2g

Table of contents

^{1}H and ^{13}C NMR spectra for new compound **1c**……………………………………………..……..S2

^{1}H and ^{13}C NMR spectra for new compound **1g**……………………………………………….S3

^{1}H and ^{13}C NMR spectra for new compound **2c**…………………………………………..…..S4

^{1}H and ^{13}C NMR spectra for new compound **2g**…………………………………………..…..S5

(2-Methoxymethoxynaphthalen-1-yl)propynoic acid naphthalen-2-yl ester (1c)

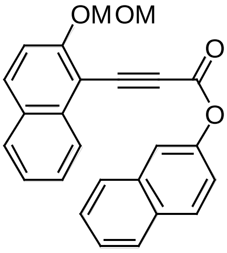

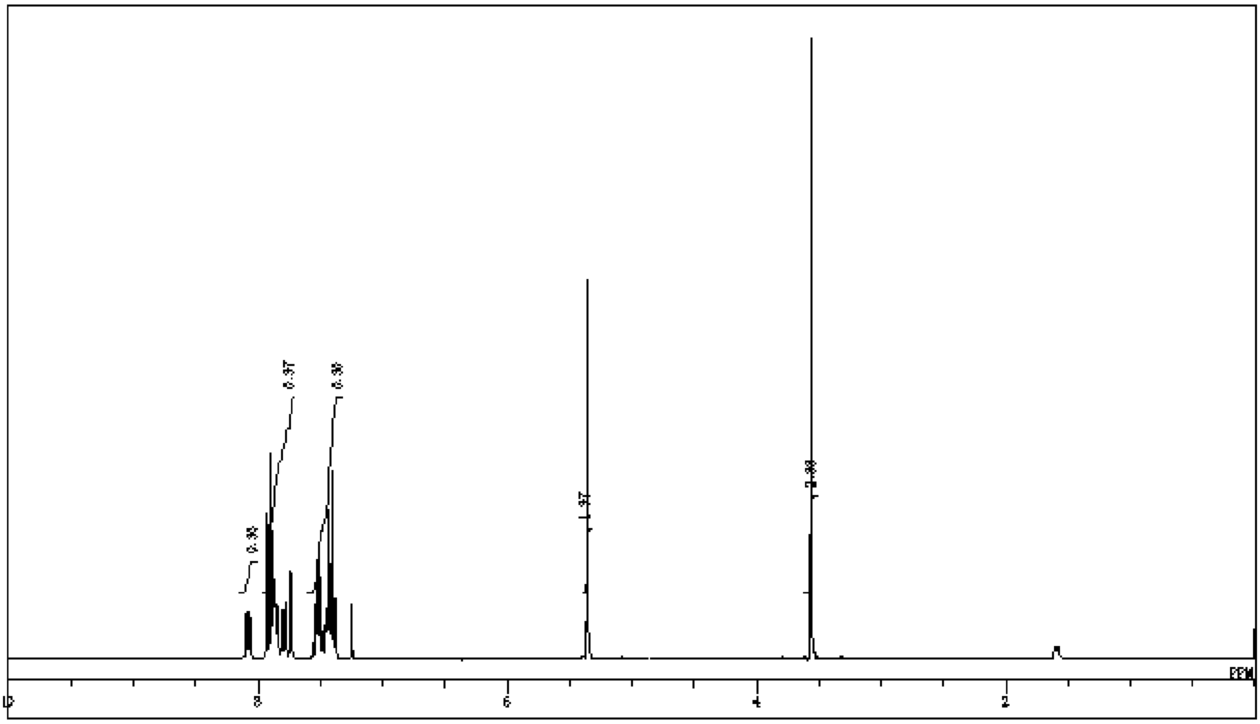

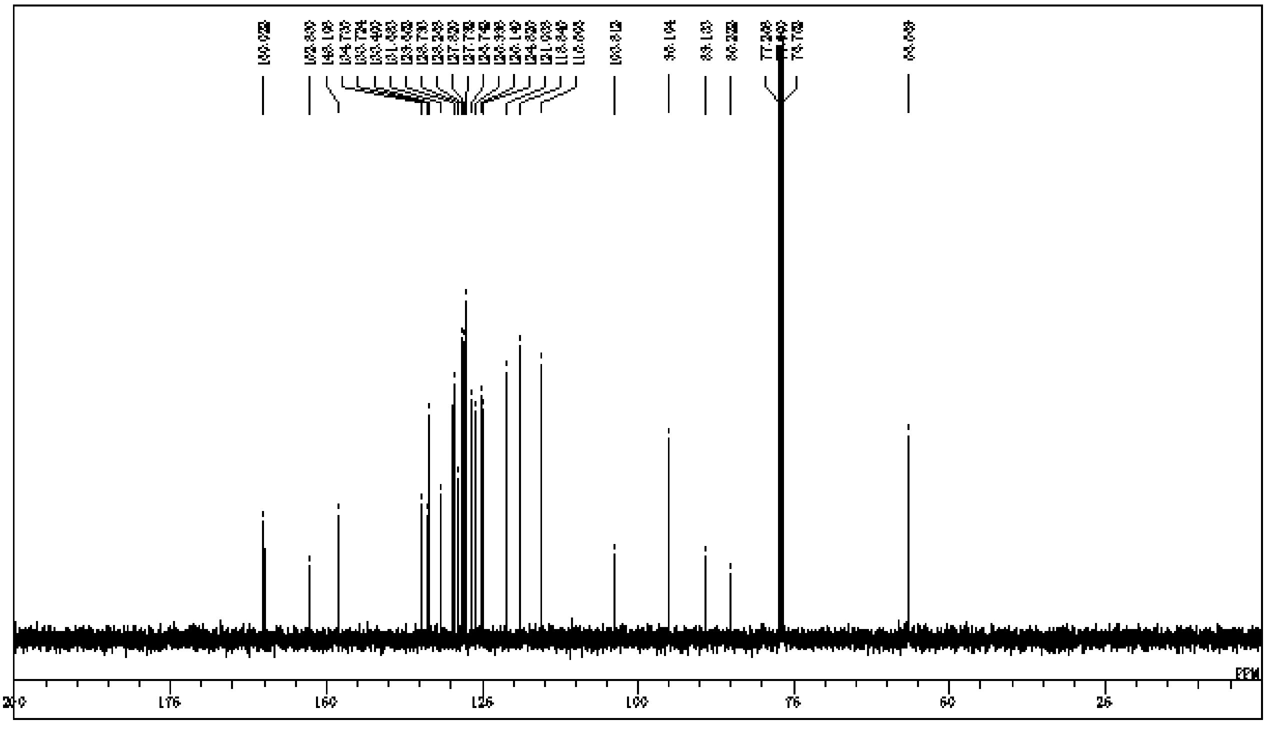

(2-Methoxynaphthalen-1-yl)propynoic acid 3,5-dimethoxyphenyl ester (1g)

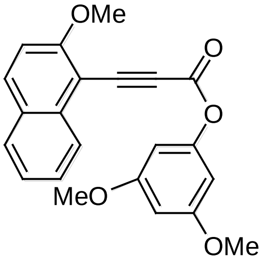

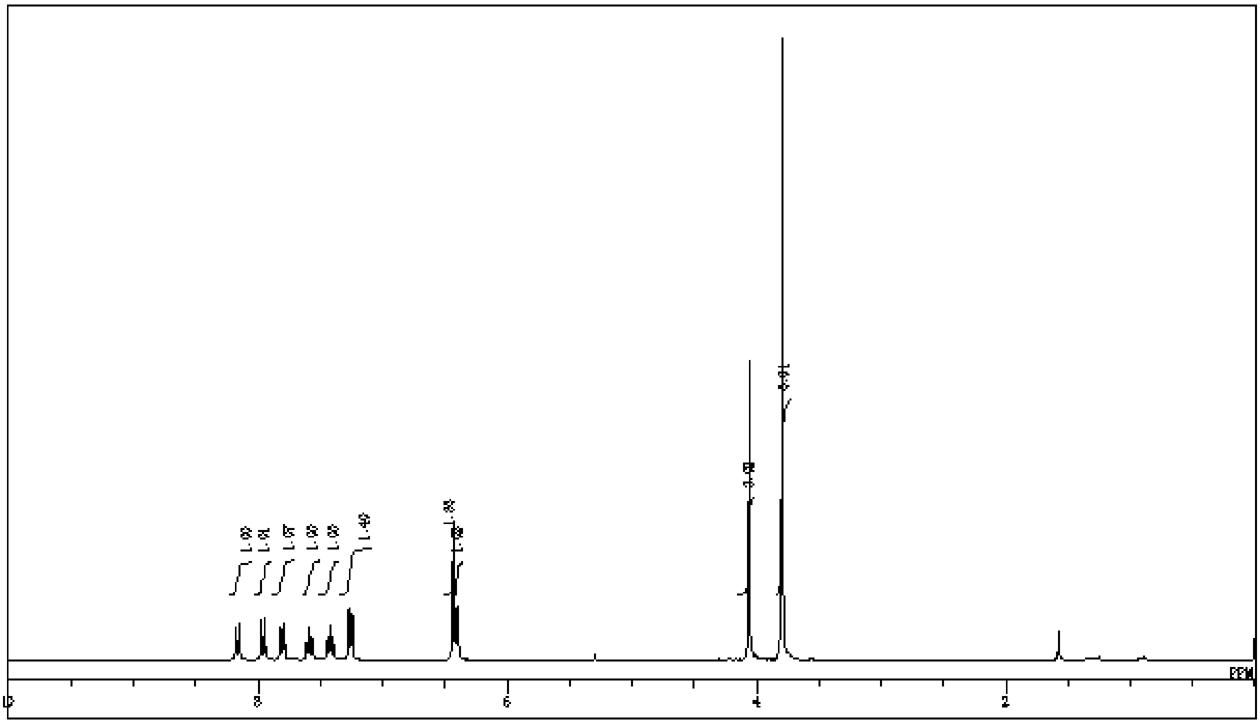

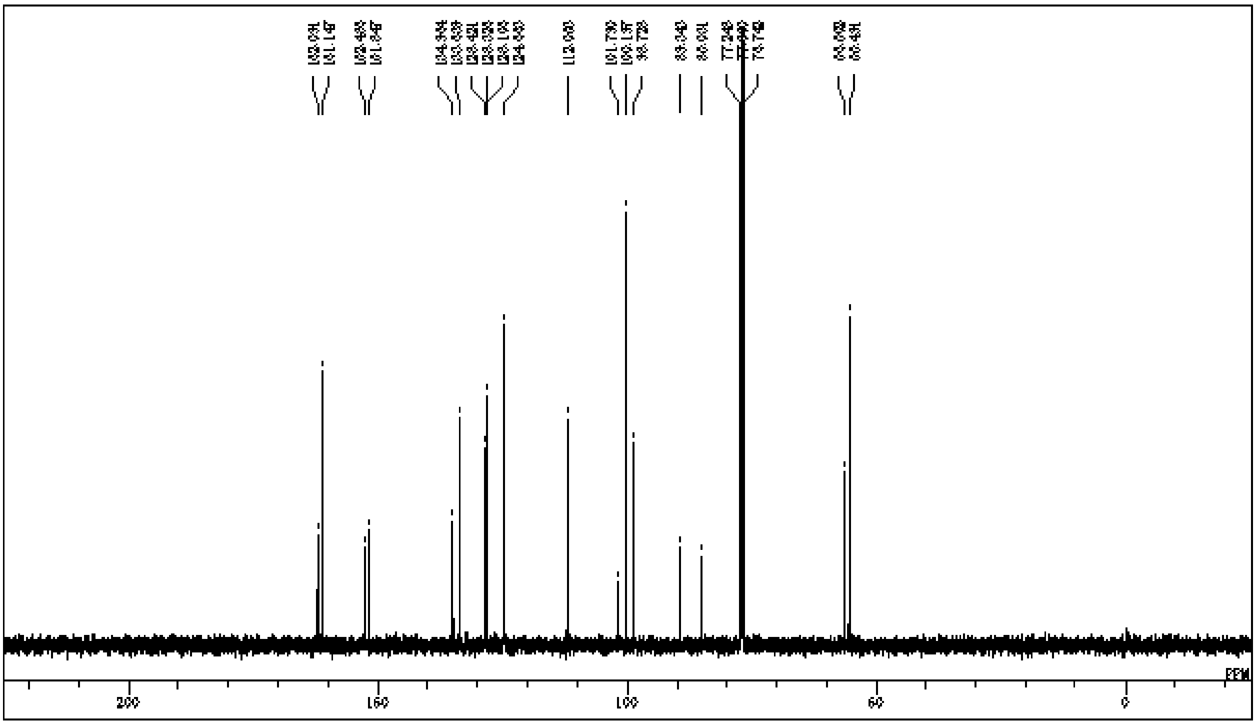

1-(2-Methoxymethoxynaphthalen-1-yl)-benzo[*f*]chromen-3-one (2c)

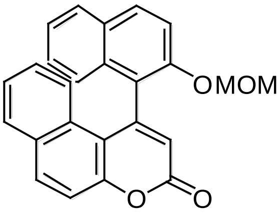

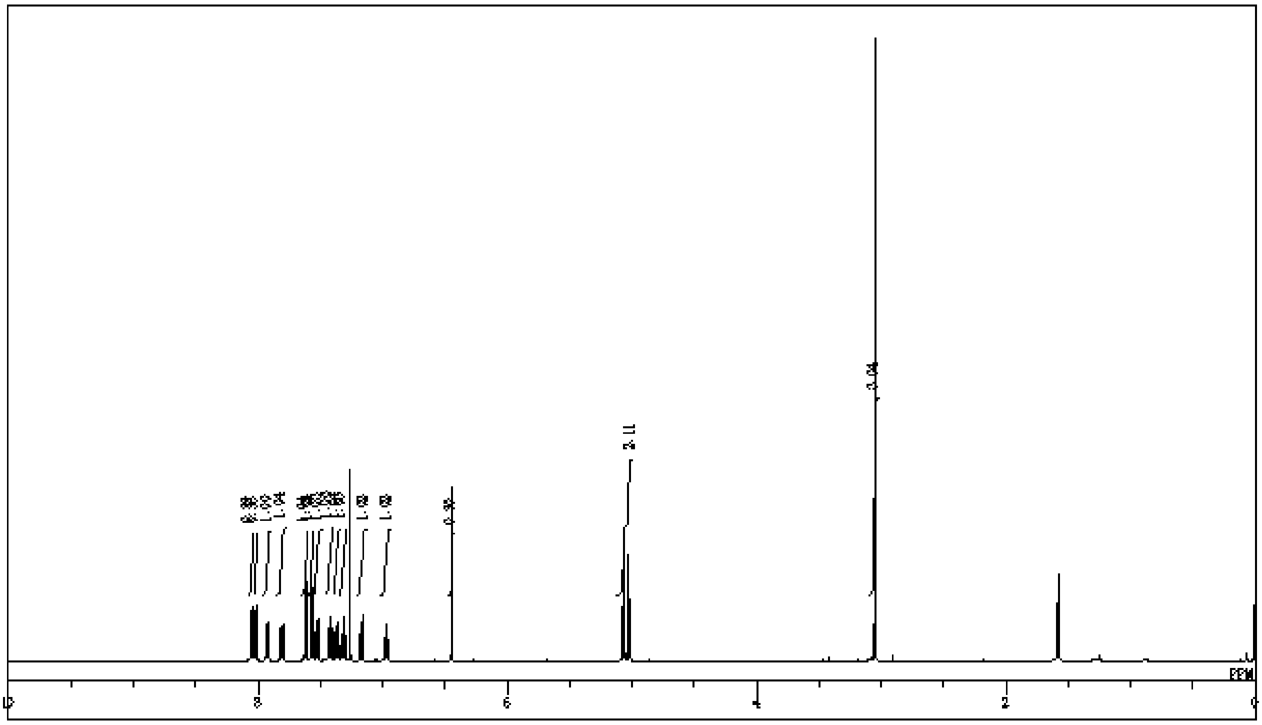

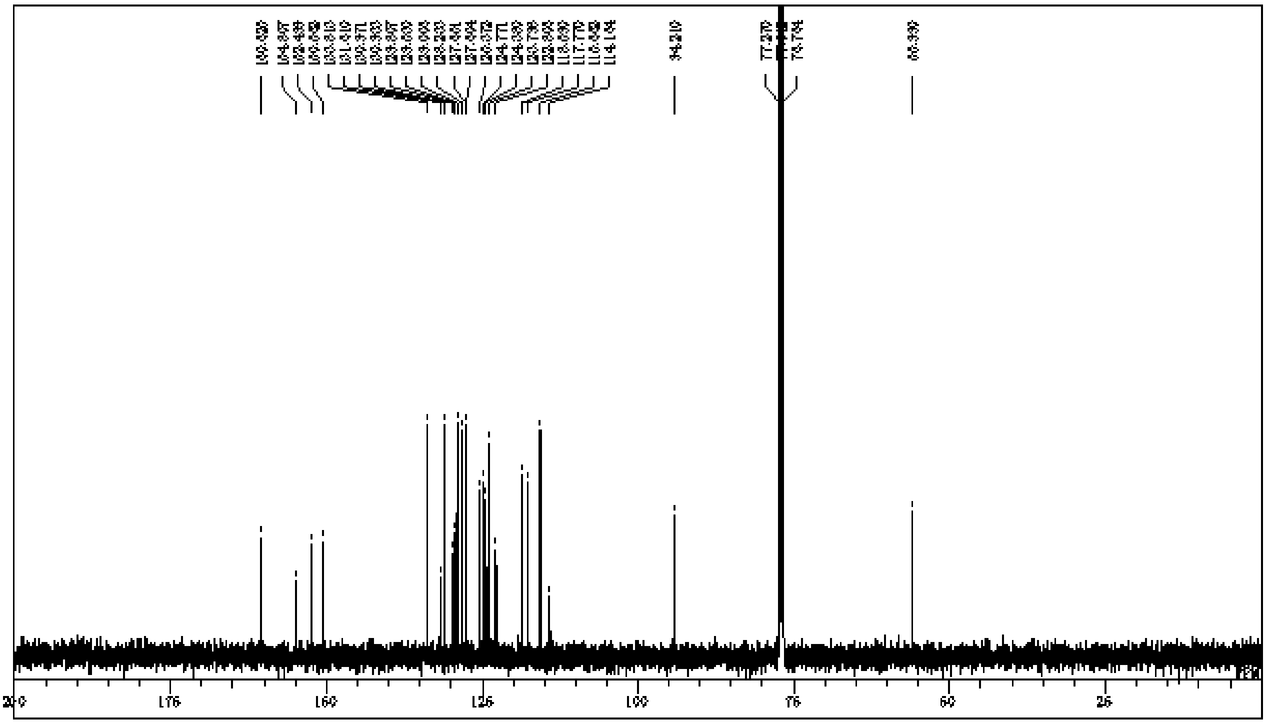

5,7-Dimethoxy-4-(2-methoxynaphthalen-1-yl)chromen-2-one (2g)

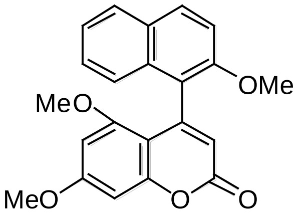

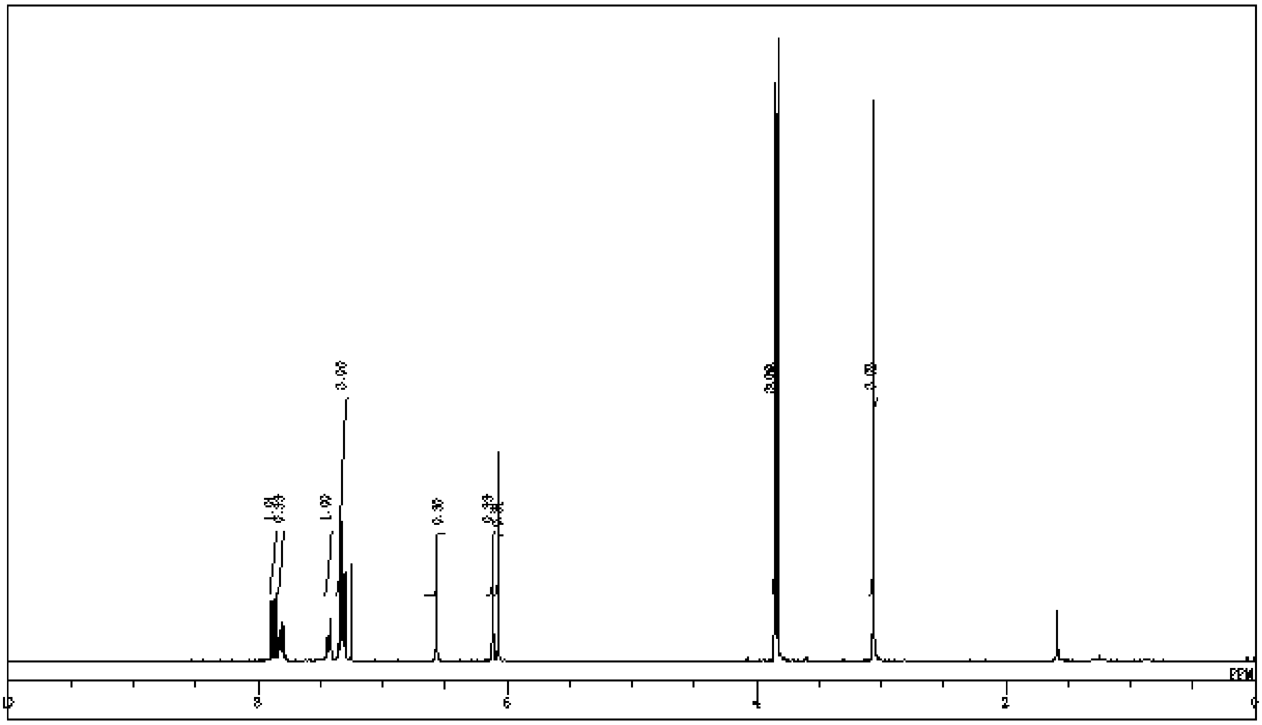

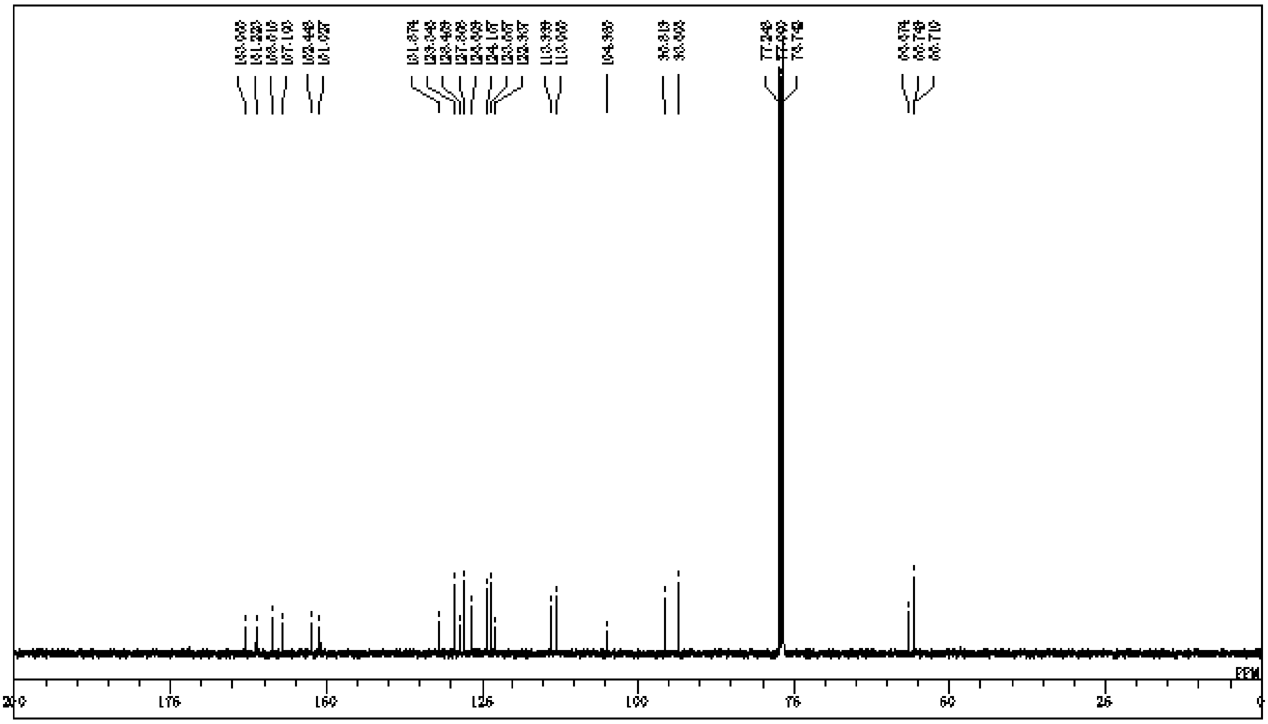

Supplement: File 1 — 1H and 13C NMR spectra for new compounds 1c, 1g, 2c, and 2g. [file Beilstein_J_Org_Chem-07-944-s001.pdf]
